# Recommendations for cerebrospinal fluid collection for the analysis by ELISA of neurogranin trunc P75, α-synuclein, and total tau in combination with Aβ(1–42)/Aβ(1–40)

**DOI:** 10.1186/s13195-017-0265-7

**Published:** 2017-06-06

**Authors:** Hugo Vanderstichele, Leentje Demeyer, Shorena Janelidze, Els Coart, Erik Stoops, Kimberley Mauroo, Victor Herbst, Cindy François, Oskar Hansson

**Affiliations:** 1 ADx NeuroSciences, Technologiepark 4, Gent, Belgium; 2 Clinical Memory Research Unit, Department of Clinical Sciences, Lund University, Malmö, Sweden; 3 IDDI, Louvain-la-Neuve, Belgium; 4 Euroimmun Medizinische Labordiagnostika, Lübeck, Germany; 5 Memory Clinic, Skåne University Hospital, Skåne, Sweden

**Keywords:** CSF, Collection, Standardization, Neurogranin, Synuclein, Tau, Amyloid, Recommendations

## Abstract

**Background:**

The pathophysiology of neurodegeneration is complex. Its diagnosis requires an early identification of sequential changes in several hallmarks in the brains of affected subjects. The presence of brain pathology can be visualized in the cerebrospinal fluid (CSF) by protein profiling. It is clear that the field of Alzheimer’s disease (AD) will benefit from an integration of algorithms including CSF concentrations of individual proteins, especially as an aid in clinical decision-making or to improve patient enrolment in clinical trials. The protein profiling approach requires standard operating procedures for collection and storage of CSF which must be easy to integrate into a routine clinical lab environment. Our study provides recommendations for analysis of neurogranin trunc P75, α-synuclein, and tau, in combination with the ratio of β-amyloid Aβ(1–42)/Aβ(1–40).

**Methods:**

Protocols for CSF collection were compared with CSF derived from subjects with normal pressure hydrocephalus (*n* = 19). Variables included recipient type (collection, storage), tube volume, and addition of detergents at the time of collection. CSF biomarker analysis was performed with enzyme-linked immunosorbent assays (ELISAs). Data were analyzed with linear repeated measures and mixed effects models.

**Results:**

Adsorption to recipients is lower for neurogranin trunc P75, α-synuclein, and tau (<10%), as compared to Aβ(1–42). For neurogranin trunc P75 and total tau, there is still an effect on analyte concentrations as a function of the tube volume. Protocol-related differences for Aβ(1–42) can be normalized at the (pre-)analytical level using the ratio Aβ(1–42)/Aβ(1–40), but not by using the ratio Aβ(1–42)/tau. The addition of detergent at the time of collection eliminates differences due to adsorption.

**Conclusions:**

Our study recommends the use of low protein binding tubes for quantification in CSF (without additives) of all relevant CSF biomarkers. Pre-analytical factors have less effect on α-synuclein, neurogranin trunc P75, and total tau, as compared to Aβ(1–42). The ratio of Aβ(1–42)/Aβ(1–40), but not Aβ(1–42)/tau, can be used to adjust for pre-analytical differences in analyte concentrations. Our study does not recommend the inclusion of detergents at the time of collection of CSF. The present results provide an experimental basis for new recommendations for parallel analysis of several proteins using one protocol for collection and storage of CSF.

**Electronic supplementary material:**

The online version of this article (doi:10.1186/s13195-017-0265-7) contains supplementary material, which is available to authorized users.

## Background

The pathophysiology of the neurodegenerative process is complex. Neurodegeneration is characterized by sequential changes observed in the brain of affected subjects, starting several decades before the onset of clinical symptoms [1]. It is clear that the field of Alzheimer’s disease (AD) will benefit from integration of algorithms, based on concentrations of individual proteins, as an aid in clinical decision-making or to improve patient enrolment in clinical trials.

The diagnosis of AD focused in the past especially on the use of proteins which have been identified in plaques and tangles, such as β-amyloid (Aβ) and tau [2]. However, other cerebrospinal fluid (CSF) proteins (e.g., synuclein, neurogranin) can reveal the presence of co-pathologies in the brain, such as Lewy bodies or loss of synapses. In this respect, identification of relevant protein profiles can further support the value of subject stratification for inclusion in clinical trials, since the biomarkers in the CSF can provide information on the nature and degree of the pathological processes in the brain of affected subjects.

CSF biomarker analysis is already integrated in (research) criteria for the diagnosis of AD [3, 4], while Aβ positron emission tomography (PET) imaging has been approved by the Food and Drug Administration (FDA) to identify subjects with ongoing amyloidopathy [5]. Efforts are on-going in Europe (European Medicines Agency (EMA)) and the United States (FDA) to qualify CSF proteins for inclusion in clinical trials [6, 7]. The performance evaluation of the in vitro diagnostic (IVD) assays will have to be performed using well-documented standard operating procedures (SOPs) for the handling of biological fluids.

Some recommendations for collection and storage of CSF for protein analysis were published previously. The standardization efforts in the field of AD focused predominantly on CSF Aβ(1–42) [8, 9]. They included the generation of reference methods [10] and the development of fully automated quantification systems [11]. Recently, it was shown that the ratio of Aβ(1–42)/Aβ(1–40) is preferred over Aβ(1–42) alone, not only because of its better clinical performance [12–14], but also because of its robustness at the (pre-)analytical level [15]. Since an optimized diagnosis will require a multi-protein approach, it is important to generate one SOP which is applicable for all selected analytes.

The present paper extends for the first time observations made for Aβ species with respect to sample handling by the inclusion of data for total tau, α-synuclein, and neurogranin trunc P75. Data were analyzed with linear repeated measures and mixed effects models. Our study provides experimental evidence that it is possible to apply the optimized SOP for Aβ isoform analysis to SOPs for other CSF proteins. It will provide an experimental basis for new recommendations to improve the collection and storage of CSF for protein analysis in the field of AD.

## Methods

### Subjects and sample collection

**Fig. 1:**
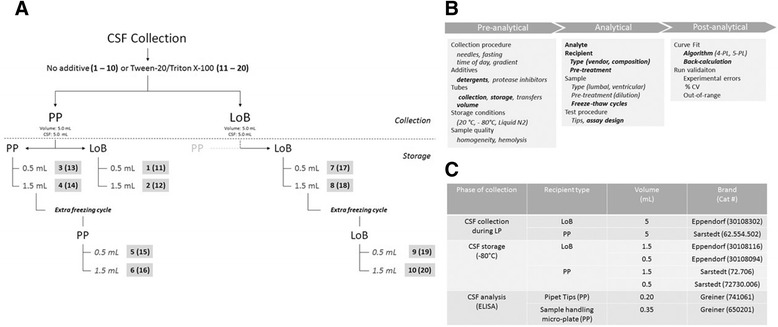
Protocols for collection and storage of CSF. **a** Schematic presentation of the collection and storage of CSF. Numbers refer to the protocol number, with (numbers 11–20) or without (numbers 1–10) addition of detergent at the time of CSF collection. **b** Variables included in the study design. **c** Recipients selected for the study. *CSF* cerebrospinal fluid, *CV* coefficient of variation, *Liquid N2 *Liquid nitrogen, *LoB* low protein binding, *LP* lumbar puncture, *PL* parameter logistic, *PP* polypropylene

CSF was collected prospectively from patients undergoing lumbar puncture (LP) due to clinical suspicion of normal pressure hydrocephalus at the Memory Clinic, Skåne University Hospital, Sweden. In these patients, 40 mL of CSF was collected as part of the clinical routine investigation. Anonymized CSF samples from 19 individuals were investigated. The detailed procedure for CSF collection and storage is shown in Fig. 1a. In addition, Fig. 1 gives an overview of the factors that were evaluated in the present study (Fig. 1b) and information on the selected recipients (Fig. 1c).

For each subject, 20 different SOPs were applied. Several vials with 5 mL of CSF were collected during the LP from the same subject in two types of tubes (low protein binding (LoB) or polypropylene (PP)) with or without additives (Tween-20 (Tw20; Panreac AppliChem, MO, USA) or Triton-X100 (TrX100; VWR Chemicals, Stockholm, Sweden)). Each tube was graduated in order to obtain the exact collection volume. A possible interference by a gradient effect was overcome by randomization of the order in which the different samples were collected. In a subset of the collection tubes, 25 μl of 10% Tw20 or TrX100 was added (final concentration 0.05% v/v). All vials were mixed by inverting them 25 times. The vials were kept at room temperature (RT) for 20 min, followed by centrifugation (2000 g, RT, 10 min). From these original tubes, CSF was transferred into microtubes (0.5 ml/1.5 ml, LoB/PP). After 1 week of storage at −80 °C, one 1.5-mL PP or LoB tube containing 1.3 mL CSF was thawed, aliquoted, and stored at −80 °C before testing. Among the tested procedures, there was no SOP using a collection LoB tube and a PP storage tube. All samples were analyzed after one freeze/thaw cycle. For the cases included in the study, CSF collection started on 23 September 2014 and ended on 2 December 2014. Sample analysis was performed in the first quarter of 2016. The measured concentrations for each analyte and subject included in the study are given in Additional file 1: Table S1.

### Assays

CSF proteins were quantified by a CE-marked enzyme-linked immunosorbent assay (ELISA) for total tau (Euroimmun, Lübeck, Germany) and research ELISAs for α-synuclein or neurogranin trunc P75 (produced in the facilities of ADx NeuroSciences, Gent, Belgium). All assays were designed with a combination of two well-characterized monoclonal antibodies (mAbs). Some details of the test procedures are described in Additional file 1: Table S2.

Before analysis, CSF samples were pipetted into pre-blocked 96-well polypropylene plates as described in detail previously [15]. The impact of the addition of detergents to calibrators and/or CSF on analyte concentrations, as measured in the current assay designs, was verified before analyzing the samples of the study.

CSF and run-validation samples (i.e., calibrators added to phosphate-buffered solutions) were analyzed in duplicate in one lab (ADx) by one operator with one lot number of each ELISA. CSF samples from each individual subject were tested on the same 96-well plate in order to limit inter-plate variability. Reported values (mean of two optical density (OD) values) were used to calculate concentrations using a four parameter logistic (4-PL) curve fit algorithm. Acceptance criteria for run validation included: (i) re-calculated values of each calibrator concentration not to exceed 15% of the nominal concentration; (ii) kit controls within pre-defined specifications; and (iii) the variability between replicates must be lower than 20%.

### Analytical performance of the neurogranin trunc P75 assay

The analytical specificity of the new neurogranin assay was verified in sandwich immuno-assay formats with synthetic peptides covering either the full length of the protein (Sequence: UniProtKB—Q92686) or peptides truncated at the amino- or carboxy-terminus. Phosphorylation at amino acid Serine 36 was included as an additional confounding factor. Several approaches were used to determine the assay selectivity, including but not limited to the replacement in the assay design of either the capture mAb or the detector mAb by a non-neurogranin-specific mAb or by the addition of proteins known to be present in CSF.

Parallelism was verified according to CLSI guideline EP06-A. Two neat CSF samples were diluted up to 1/10 (in the working range of the assay) with sample diluent and tested in quadruplicates. Concentrations were calculated taking into account the dilution factor.

Precision was evaluated using native CSF samples.

### Analytical performance of the α-synuclein assay

The specificity of the assay for the detection of α-synuclein (as compared to β-, or γ-) was verified with recombinant proteins (Source: rPeptide, Fremont, California, USA). Parallelism was verified according to EP06-A. Four native CSF samples were serially diluted with sample diluent up to 1/20 and tested in duplicate. Concentrations were calculated taking into account the dilution factor. Precision was evaluated using native CSF samples.

### Analytical performance of the total tau assay

The analytical performance characteristics for the total tau assay have been summarized previously [16].

### Comparison of adsorption rate in function of analyte or ratios of analytes

The relative difference in adsorption rate between analytes (individual proteins, ratio of proteins) were visualized by plotting the mean relative differences for all subjects when a comparison was made between the selected reference protocol (Protocol 7) and the protocol that always resulted in the highest relative difference (Protocol 6) or between the reference protocol and a protocol including CSF collection in PP tubes (Protocol 1).

### Statistical analysis of the CSF SOP experiment

The SOP experiment provided repeated measures data. The different measurements recorded on CSF collected from the same subject form a multivariate response. Measurements made with samples of the same subject are correlated, while measurements between different subjects are considered independent (with CSF of different subjects tested in different assay runs). More details on the analysis are described in reference [15] and Additional file 1: Figure S1. For each analyte, the impact of the analyte concentration on the calculated relative difference was verified by regression analysis.

## Results

### Assay analytical performance

An overview of the performance characteristics of each immuno-assay is essential to qualify the value of the results obtained in this study on standardization of the process for collection and storage of CSF. Part of the assay performance characteristics for the neurogranin trunc P75 and α-synuclein assay are described in Additional file 1: Figure S2 and Figure S3, respectively.

**Fig. 2:**
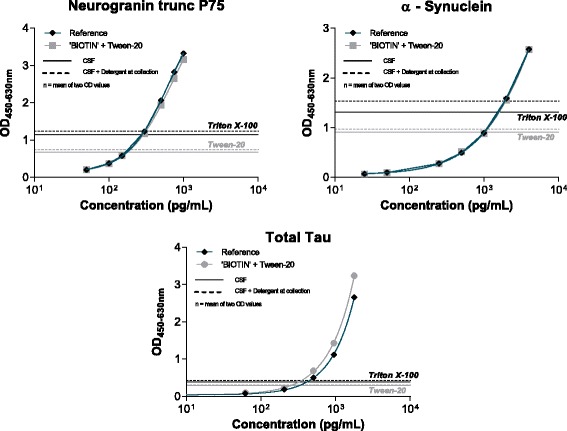
Effect of the addition of 0.05% detergent to the BIOTIN component (= biotinylated antibody) on calibration curves and samples in the neurogranin trunc P75, α-synuclein, and total tau. A representative calibration curve is presented. Each calibrator point is the mean of duplicate optical density (*OD*) values. *Horizontal lines* represent the mean OD values derived from all cerebrospinal fluid (*CSF*) samples as a function of the relevant collection protocol

The addition of detergent (Tw20, Trx100) in the assay during sample incubation affects the outcome of the assay (= concentration) differently as a function of the analyte, the detergent, or OD values for calibrators and samples (Fig. 2). For calibrators, no effect on OD values were shown for neurogranin trunc P75 and α-synuclein, while OD values were higher for tau calibrators added to a buffer in which Tw20 was added. For CSF, slightly higher OD values were noted after the addition of detergent into the neurogranin trunc P75 and α-synuclein assay, but not for the tau assay. For α-synuclein, we observed a possible difference in function of the selected detergent type.

In order to standardize the measurement of the analytes in the study, and since our study design included different CSF compositions (no detergent, Tw20, TrX100), we performed the simultaneous incubation of the sample (CSF, calibrator, kit controls) with biotinylated antibodies dissolved in a buffer containing 0.05% (final concentration) Tw20. We observed no (major) difference in function of the selected detergent.

### Optimization of the CSF collection and storage procedure for neurogranin trunc P75, α-synuclein, and tau

**Fig. 3:**
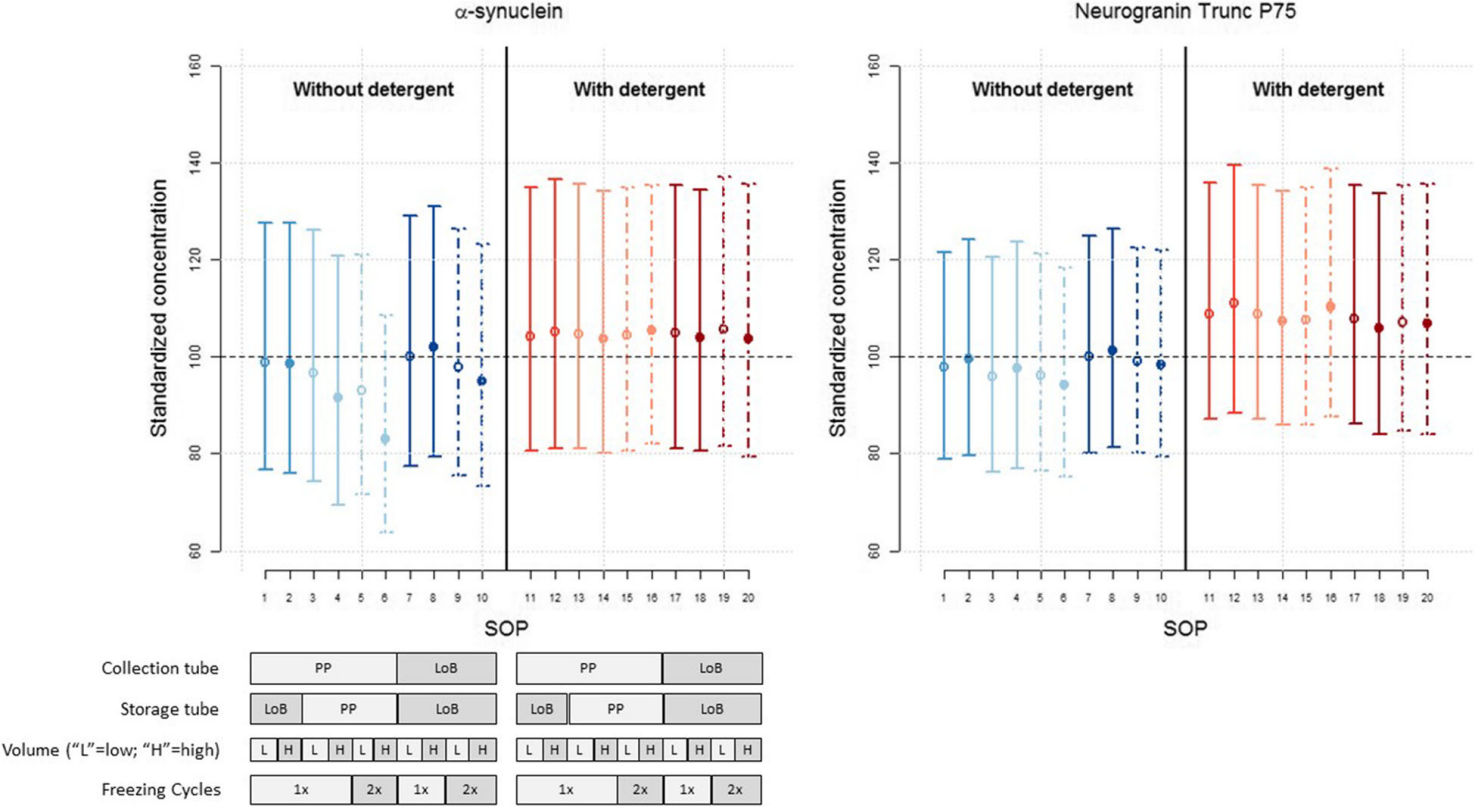
Standardized mean concentration for each CSF protocol and analyte. Standardized mean concentrations (±95% confidence intervals) for each CSF protocol, ordered from the highest to the lowest standardized concentration using protocol 7 as the reference. Numbers on the *x* axis correspond to the numbers described in the collection protocol (Fig. 1). *Blue* colors are linked to procedures without detergent, *red* colors with the addition of detergent at the time of collection. The *line* style is selected as a function of the collection procedure. Effect sizes and *p* values are described in Additional file 1: Table S3. *LoB* low protein binding, *PP* polypropylene, *SOP* standard operating procedure

Figure 3 shows standardized mean concentrations for each CSF protocol, ordered as a function of the protocol number. Procedure 7 was considered previously as a possible reference protocol for further analysis. This was based on results obtained for the ratio Aβ(1–42)/Aβ(1–40). Details of the pair-wise differences between SOPs and *p* values are described in Additional file 1: Table S3. When looking at differences among procedures without the addition of detergent to CSF at the time of collection (Fig. 3; SOPs 1–10, left-hand side, blue scales), procedure 8 resulted in the highest observed mean concentration, followed by procedure 7. Both SOPs 7 and 8 use LoB collection and storage tubes and have no additional freeze-thaw cycle. For all analytes, the lowest observed mean concentration was with SOP 6 that combines a PP collection tube, a high volume PP storage tube (= higher percentage of CSF versus recipient surface area), and an additional freeze/thaw cycle. All in all, the differences among SOPs are modest, with the largest effects seen for neurogranin. The difference in mean concentration between SOP 7 and SOP 6 (the maximal difference, lower limit, upper limit) was estimated to be a decrease of 16.9% (−20.2%, −13.5%) for neurogranin (*p* < 0.0001), 5.7% (−9.4%, −1.7%) for α-synuclein (*p* = 0.005), and 8.8% (−11.7%, −5.8%) for total tau (*p* < 0.001). In the presence of detergent at the time of collection (Fig. 3; SOPs 11–20, right-hand side, red color), there was only a slight decrease of 4.3% (−7.0%, −1.4%) for total tau (*p* = 0.004) between SOP 11 and SOP 17. All other differences were not significant.

In addition, it was calculated that the observed differences of protocol 6 as compared to the reference method (= protocol 7) was not dependent on the protein concentration in the CSF.

**Fig. 4:**
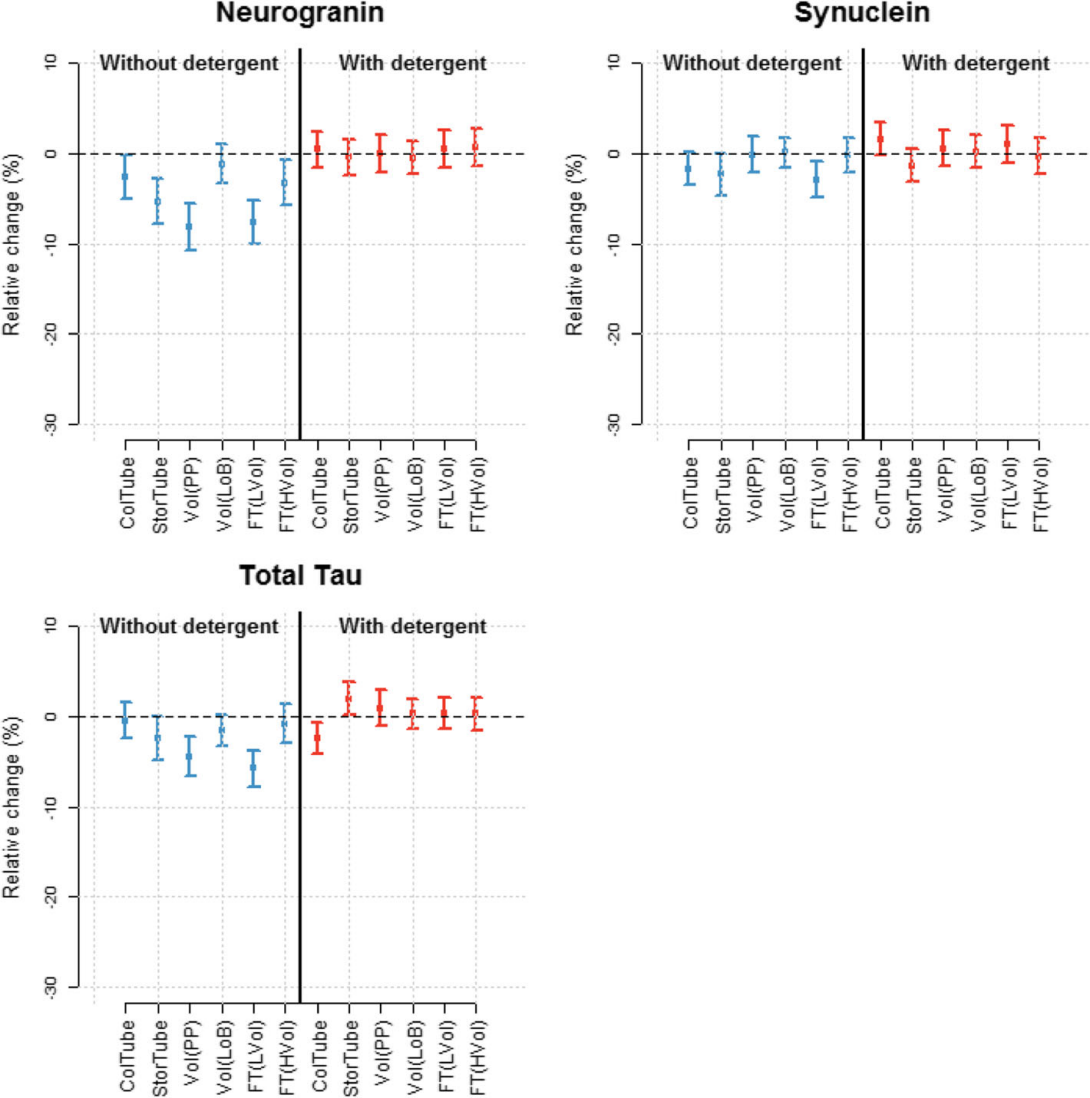
Optimization of CSF collection procedures. Effects of different factors. Results are shown as mean ± 95% confidence intervals for each factor in the analysis. *ColTube* collection tube, *FT* freeze/thaw, *H* high, *L* low, *LoB* low binding, *PP* polypropylene, *StorTube* storage tube, *Vol* volume

In the second analysis (Fig. 4; Additional file 1: Table S4), the effects of the different procedural factors and their relationship were estimated for each analyte. In the absence of detergent (SOPs 1–10), the same pattern of effects was revealed for neurogranin and total tau, albeit with somewhat larger effects for neurogranin. For all analytes, the collection of CSF in PP tubes resulted in lower concentrations compared to collection in LoB tubes, amounting to a decrease of 2.7% (−0.3, −5.1%; *p* = 0.030), 1.7% (0.1, −3.5%; *p* = 0.063), and 0.5% (1.5, −2.5%; *p* = 0.574) for neurogranin, α-synuclein, and total tau, respectively.

The transfer to a PP storage tube had a larger effect and decreased the concentration on average by 5.3% (−7.8%, −2.8%; *p* = 0.0004), 2.4% (−4.7%, 0.0%; *p* = 0.052), and 2.5% (−4.8%, −0.1%; *p* = 0.043) for neurogranin, α-synuclein, and total tau, respectively.

Storage tube volume had no effect on the measured α-synuclein concentrations. For neurogranin and total tau, the effect of tube volume was dependent on the storage tube type, with larger and significant effects for PP tubes. For neurogranin, a large PP tube volume decreased the concentration by 8.1% (−10.7%, −5.5%; *p* < 0.0001) whereas for a LoB storage tube the estimated decrease was only 1.2% (−3.4%, 1.0%; *p* = 0.275). For total tau, a smaller volume of PP storage tube decreased the concentration significantly by 4.5% (−6.7%, −2.2%; *p* = 0.001) whereas for a larger volume of the storage tube, the estimated decrease was only 1.6% (−3.4%, 0.2%; not significant (*p* = 0.073)).

For all three analytes, the effect of an additional freeze-thaw cycle was dependent on the storage tube volume. The effect was more pronounced for tubes with a larger volume. For low volume tubes, the concentration was significantly decreased by an additional freeze-thaw cycle only for neurogranin, amounting to a decrease by 3.4% (−5.8%, −0.8%; *p* = 0.013). For α-synuclein and total tau, estimated differences in concentration after freezing were below 1%. For the larger storage tubes, the effect of an additional freeze-thaw cycle was a significant decrease in concentration of 7.7% (−10.1%, −5.3%; *p* < 0.0001) for neurogranin, 3.0% (−4.9%, −1.0%; *p* = 0.0056) for α-synuclein, and 5.8% (−7.9%, −3.8%; *p* < 0.0001) for total tau.

With the addition of detergent at the time of collection of CSF (SOPs 11–20), measured concentrations increased for all three analytes ( Fig. 4; right-hand side, red scales) with all standardized mean concentrations for SOPs 11 to 20 being above 100. The differences among SOPs, on the other hand, were largely attenuated. For neurogranin and α-synuclein, none of the factors affected the analyte levels significantly. For total tau, the collection and storage tube type affected the concentration levels significantly even in the presence of detergent. The use of a PP collection tube decreased the total tau concentration by 2.5% (−4.3%, −0.8%; *p* = 0.008) and the use of a PP storage tube increased the concentration by 1.9% (0.1%, 3.7%; *p* = 0.036) compared to a LoB tube.

### Comparison of adsorption rate as a function of analyte

**Fig. 5:**
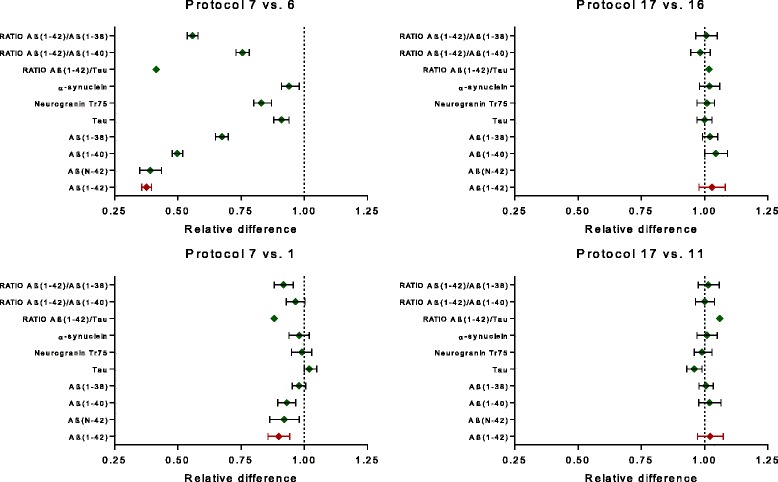
Visualization of difference in adsorption rate between CSF analytes or ratios of analytes. Results are shown as mean ± 95% confidence intervals. Note: results for β-amyloid (*Aβ*) have been presented in [15]

The overall difference in adsorption rate of individual proteins or ratio of CSF proteins is shown in Fig. 5. A comparison was made between protocol 7 (= the reference protocol) and protocol 6 or protocol 1 and as a function of the inclusion of detergent at the time of collection of CSF.

In the absence of detergent at the time of collection (SOPs 1–10), the biggest difference with collection in LoB tubes is obtained with Aβ(1–42), followed by Aβ(1–40) and Aβ(1–38) (data modified according to [15]). Results for Aβ(1–42) and Aβ(*N*–42) are identical (the latter is quantified by replacement in the assay design of mAb 3D6 (specific for Aβ1) by mAb 1A1 (kindly provided by Eli Lilly; the mAb is specific for Aβ*N*)). Changes in concentration of neurogranin as a function of collection protocol are lower than for the Aβ isoforms. Effects are even lower for tau or α-synuclein, but are still significant. Adsorption problems linked to the use of Aβ(1–42) can be normalized in part by the ratio Aβ(1–42)/Aβ(1–40), but not by using the ratio Aβ(1–42)/tau.

In the presence of detergent at collection (SOPs 11–20), all analytes or ratio of analytes show comparable results to those obtained with the reference protocol.

## Discussion

This study describes the effects of pre-analytical variables on the quantification in CSF of several proteins which are directly involved in the neurodegenerative process. The recommended protocol as described in the study is applicable for each selected protein in the CSF biomarker panel. Our study results will speed-up the integration of biomarker panels into clinical trials.

Recent late-stage failures of clinical trials in the field of AD will force the community to identify better tools to select drug candidates which might become successful as disease-modifying drugs. When using algorithms including concentrations of a number of different biomarkers (e.g., synapse proteins, tau, Aβ isoforms) for decision-making, one needs to understand how each individual protein will be affected by changes in (pre-)analytical conditions. For example, Bjerke et al. [17] reported that only native CSF without additives is commutable for multiple method comparisons, including mass spectrometry. In addition, the selection of the matrix type for use as a quality control sample in the assay is important [18], while the effect of changes in biomarker concentrations on clinical decision-making must be documented for each analyte [19]. If needed, statistical approaches are available for harmonization of results between study designs or between sample processing procedures [20].

The SOP experiments were analyzed with linear repeated measures and mixed effects models. These analysis methods properly handle the correlations between the measurements and enable the combination of measurements from all samples to derive one average effect. An important added value of this versatile statistical approach is that it allows the breakdown of the observed differences among SOPs into effects caused by different factors and their interplay.

Our data show that CSF concentrations of total tau, α-synuclein, and neurogranin trunc P75 are less affected than Aβ isoforms by changes in collection protocols. Although some statistical differences were detected in some cases, we consider them considerably lower (<10.0% difference in protein concentration versus the reference protocol) as compared to what was published previously for Aβ(1–42) (>50% difference in concentration) [15]. The presence of detergent (TrX100 or Tw20) at the time of collection normalizes the results between study protocols. Partial normalization of protocol differences is obtained when using the ratio of Aβ(1–42)/Aβ(1–40), but not by using the ratio Aβ(1–42)/Tau. Thus, although the combination of Aβ(1–42)/tau has been suggested as an alternative for Aβ(1–42) when comparison has to be done with amyloid PET imaging, this combination cannot solve the pre-analytical aspects. One needs to take into account analytical and clinical aspects of the biomarker analysis when statements are made for the use of a specific protein combination for a specific context of use.

Publications on (pre-)analytical standardization for analysis in CSF of neurogranin or α-synuclein are limited. Kruse et al. [21] reported data on handling of CSF using another commercially available α-synuclein ELISA. In contrast to our study, they did not observe an effect on the aliquot volume, which might be related to our experimental protocol or the selected sample types. For the above-mentioned assay, there was still a problem of lot-to-lot variability [22].

Our results provide experimental evidence that it is possible to extend the panel of CSF proteins, using the same protocol for collection and storage as for the classical CSF proteins, with synapse proteins. Synapse loss correlates with cognitive decline and is more associated within certain areas of the brain with the degree of dementia as compared to plaques and tangles [23]. Neurogranin, a calmodulin-binding postsynaptic neuronal protein, is abundantly expressed in perikaryal and dendritic cytoplasm. It is present as a carboxy-terminal modified peptide that can be measured in human brain [24], CSF [25–27], and plasma [27]. α-Synuclein has a function in synaptic plasticity, brain lipid metabolism, and regulation of vesicle trafficking [28]. The protein is localized in the pre-synaptic neuronal terminals. α-Synuclein is the main component of Lewy bodies and is highly abundant in erythrocytes and platelets. The protein might be released from cells after freezing [29].

The visualization of adsorption rates as a function of procedure and analyte will allow researchers to focus on the most important factors during standardization of collection and storage of CSF. The optimal protocol for quantification of Aβ isoforms can be easily integrated into protocols for other CSF analytes. However, some factors, such as the storage tube or the number of freeze-thaw cycles, can still affect the concentration determination in CSF, especially when the volume of sample per tube is taken into account. The impact of these differences on individual patient management or clinical decision-making will have to be documented in future diagnostic studies since these markers are not as integrated yet into the field of neurodegeneration as the tau and Aβ proteins.

The addition of detergents at the time of collection of CSF can reduce the observed differences in adsorption rate as a function of the collection and storage protocols. However, this will result in a higher order of complexity for the already required detailed SOPs for the collection of CSF. The latter is related to the selected detergent and its in-process quality control, the need to optimize the working concentration of the detergent as well as how the detergent is added to the sample (volume/volume ratio), the time of presence in the sample before freezing, and the need to standardize the collected volume of sample per tube. Studies will have to verify whether samples with detergents are still commutable with results obtained with the available reference method for Aβ(1–42) [10] or whether samples with detergent react in the same way as neat biological samples (as a function of time, temperature, shaking, freezing) with the antibodies in the assay design.

There are some limitations to our study protocol. The study was performed with two different detergents. Since no significant differences were found between the selected detergents, data were analyzed assuming the detergents had an identical effect. The study was performed with an ad-hoc design that has limitations to study the interplay among several factors. Among the tested procedures, there was no protocol using a collection LoB tube and a PP storage tube. As such, the reported effect of the storage tube type should be interpreted as the effect after collection in a PP tube. The additional freeze/thaw cycle was only performed in protocols with the same collection and storage tube type. Optimization of the SOPs for collection and storage of CSF was performed using one specific assay format and test procedure.

Standardization of pre-analytical and analytical variables, including the development of fully automated assays and the definition of global cut-offs, are essential to enable a path for success for CSF neurogranin and α-synuclein in the future.

## Conclusions

The study recommends the integration of the following topics into new guidelines for collection of CSF: use low binding recipients during sample handling; limit dead volume in tubes; use one collection protocol for analysis of Aβ(1–42)/Aβ(1–40) with other CSF analytes; do not include detergent at the time of collection of CSF. Taking into account their clinical value, the recommendations provided in the present paper will be able to speed-up the worldwide standardization and integration of CSF AD biomarker protein panels.

## Additional file

Additional file 1: Table S1. Concentrations (pg/mL) of neurogranin trunc P75, α-synuclein, and total tau in the CSF samples included in the study. **Table S2.** Biomaterials and test procedure. **Table S3.** Optimization of CSF collection procedures. Standardized mean concentrations for each CSF SOP, effect sizes, and *p* values. **Table S4.** Optimization of CSF collection procedures. Standardized mean concentrations for each factor, effect sizes and *p* values. **Figure S1.** Statistical analysis of the CSF SOP experiment. **Figure S2.** Analytical performance data for the neurogranin trunc P75 ELISA. **Figure S3.** Analytical performance data for the α-synuclein ELISA. (DOCX 192 kb)

## Abbreviations

AD: Alzheimer’s disease
Aβ: β-amyloid
CE: European Commission
CSF: Cerebrospinal fluid
ELISA: Enzyme-linked immunosorbent assay
EMA: European Medicines Agency
FDA: Food and Drug Administration
IVD: In vitro diagnostic
LoB: Low protein binding
LP: Lumbal puncture
mAb: Monoclonal antibody
OD: Optical density
PET: Positron emission tomography
PL: Parameter logistic
PP: Polypropylene
RT: Room temperature
SOP: Standard operating procedure
trunc: Truncated
TrX100: Triton-X100
Tw20: Tween-20
v/v: Volume to volume ratio
