# Supplementary material for: Recommendations for cerebrospinal fluid collection for the analysis by ELISA of neurogranin trunc P75, α-synuclein, and total tau in combination with Aβ(1–42)/Aβ(1–40)
Source: Alzheimers Res Ther. 2017 Jun 6;9:40. doi: 10.1186/s13195-017-0265-7 (PMC5461747; doi:10.1186/s13195-017-0265-7)
Supplement: Additional file 1: Table S1. — Concentrations (pg/mL) of neurogranin trunc P75, α-synuclein, and total tau in the CSF samples included in the study. Table S2. Biomaterials and test procedure. Table S3. Optimization of CSF collection procedures. Standardized mean concentrations for each CSF SOP, effect sizes, and p values. Table S4. Optimization of CSF collection procedures. Standardized mean concentrations for each factor, effect sizes and p values. Figure S1. Statistical analysis of the CSF SOP experiment. Figure S2. Analytical performance data for the neurogranin trunc P75 ELISA. Figure S3. Analytical performance data for the α-synuclein ELISA. (DOCX 192 kb) [file 13195_2017_265_MOESM1_ESM.docx]

**RECOMMENDATIONS FOR CSF COLLECTION FOR THE ANALYSIS BY ELISA OF NEUROGRANIN TRUNC P75, α-SYNUCLEIN, AND TOTAL TAU IN COMBINATION WITH Aß(1-42)/Aß(1-40).**

Hugo Vanderstichele^1*^, Leentje Demeyer^1^, Shorena Janelidze^2^, Els Coart^3^, Erik Stoops ^1^, Kimberley Mauroo^1^, Victor Herbst^4^, Cindy Francois^1^, and Oskar Hansson^2,5^

***Affiliations***

^1^ ADx NeuroSciences, Gent, Belgium

^2^ Clinical Memory Research Unit, Department of Clinical Sciences, Malmö, Lund University, Sweden

^3^ IDDI, Louvain-la-Neuve, Belgium

^4^ Euroimmun Medizinische Labordiagnostika, Lübeck, Germany

^5^ Memory Clinic, Skåne University Hospital, Sweden

* Corresponding author

***SUPPLEMENTARY MATERIALS***

Table S1 Concentrations (pg/mL) of neurogranin trunc P75, α-synuclein, and total tau in the CSF samples included in the study.


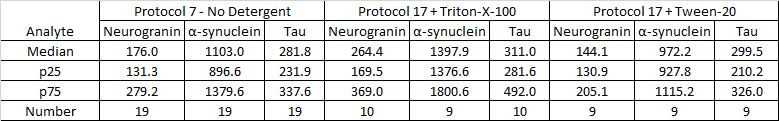
Abbreviations: p25/p75 = percentile


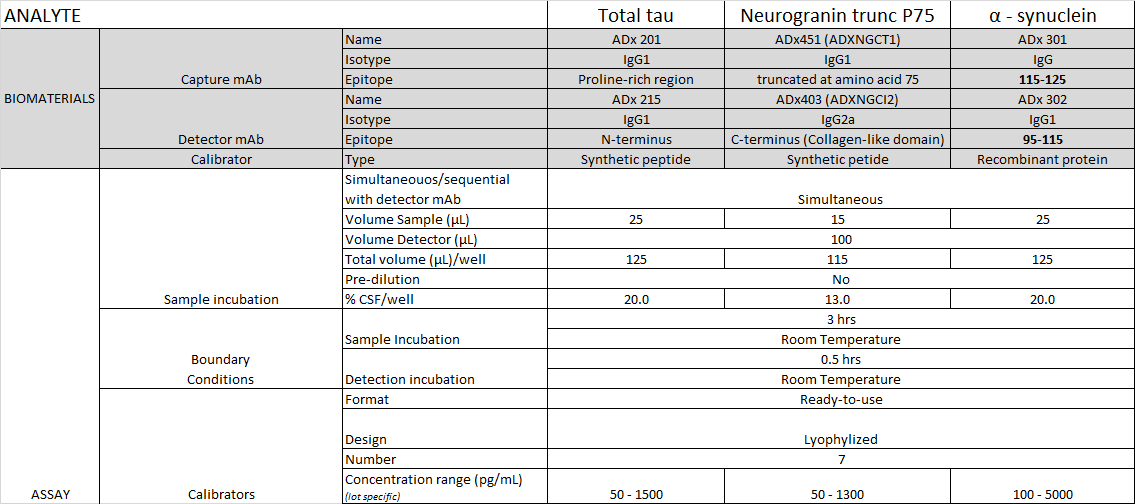
Table S2 Biomaterials and test procedure.

Note: room temperature is defined as ± 18 - 30°C.

Table S3
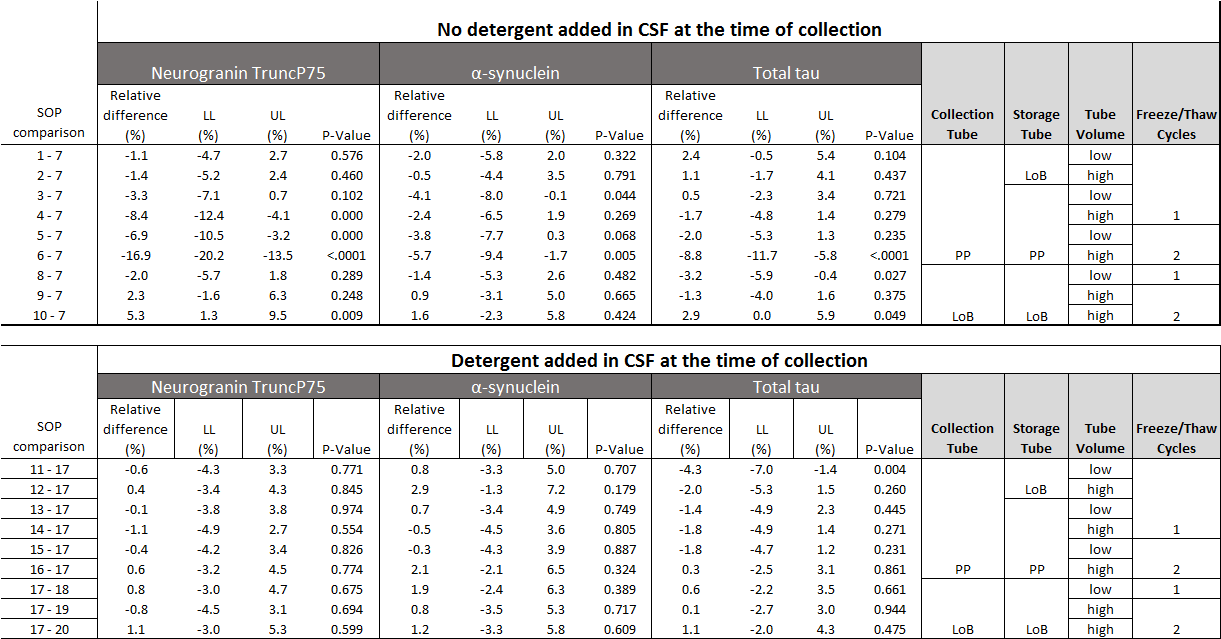
 Optimization of CSF collection procedures. Standardized mean concentrations for each CSF SOP, effect sizes, and p-values.

Abbreviations: LL = lower limit; LoB = (protein) low binding; PP = polypropylene; UL = upper limit; SOP = standard operating procedure.

Table S4. Optimization of CSF collection procedures. Standardized mean concentrations for each factor, effect sizes and p-values.


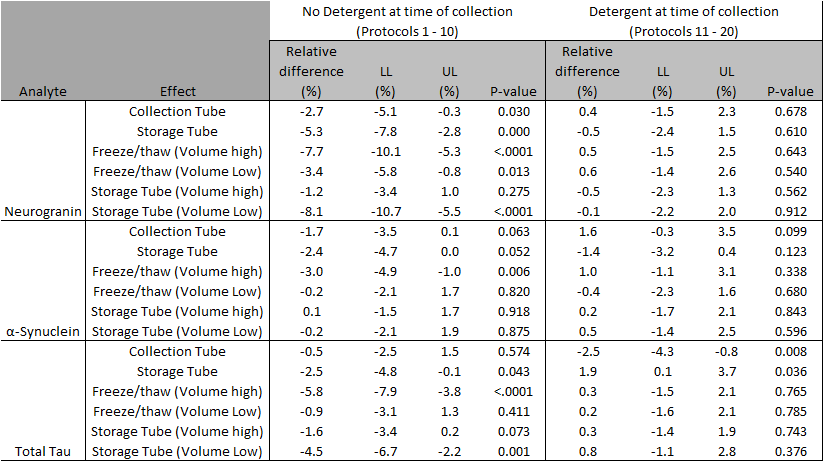


Abbreviations: LL = lower limit; UL = upper limit.

Figure S1. Statistical analysis of the CSF SOP experiment

The SOP experiment provided repeated measures data. The different measurements recorded on CSF collected from the same subject form a multivariate response. Measurements made with samples of the same subject are correlated, while measurements between different subjects are considered independent (with CSF of different subjects tested in different assay runs).

Concentrations were log-transformed prior to modeling. Correlations among measurements made on the same subject were modeled by introducing a random subject effect and through the specification of different covariance structures. Restricted maximum likelihood (REML) estimation was used to compare the models with different approaches to variance modeling but the same fixed-effects model. For all experiments, the correlated errors model (without random effect) with heterogeneous compound symmetry covariance structure was selected (based on Akaike and Bayesian information criteria). This model imposes the same pair-wise correlations among all measurements on the same subject but allows for heterogeneous variances among subjects. Model fit was approved based on residual diagnostics. The final models were rerun with ML estimation and Kenward-Roger approximation to the degrees of freedom for inference on fixed effects.

A first model was fitted to study the differences in concentrations among the 20 SOPs. This model contained only the fixed 20-level factor “CSF-SOP”. Results were reported as mean (over all subjects) standardized concentrations for each SOP together with a 95% confidence interval (CI). Concentrations were standardized by dividing the mean SOP-specific concentrations by the mean concentration of the reference procedure (SOP 7). A standardized concentration of 110 (90) means a 10% increase (decrease) in concentration compared to the reference procedure.

A second model was fitted to further evaluate the impact of the different SOP factors. The model contained the main effects of the factors collection (first tube) and storage (second tube) recipient, the volume of the storage tube, an additional freeze/thaw cycle and the addition of detergent, together with interactions of detergent with each of 4 other factors and 2 three-way interactions (for detergent, freeze-thaw condition and storage tube and for detergent, volume and type of storage tube). The reported effects of a factor are relative changes (%) in concentration, keeping the other factors constant. For instance, the estimated effect of the collection tube is the expected percent difference in concentration when CSF of the same subject is collected in PP compared to LoB collection tubes, but transferred to the same storage tube of the same volume, with the same number of freeze/thaw cycles.

Figure S2. Analytical performance data for the Neurogranin Trunc P75 ELISA

***Specificity***. The neurogranin Trunc P75 ELISA is specific for neurogranin proteins ending at position 75. Full-length proteins or proteins truncated at position 76 are not detected by the assay. There is no direct interference in immunoreactivity when peptides are phosphorylated at position Ser36 (data not shown). The analytical specificity of the assay is defined by the capture mAb, AD451 (ADxNGCT1).

***Selectivity.*** Immunoreactivity is absent in positive commercial CSF (n=10, Biomnis, France) when either the capture or the detector mAb was replaced by a non-neurogranin binding mAb. Addition of collagen Type I, III, and IV proteins did not interfere in the assay (published observations).

***Parallelism.*** Dilution of neat CSF samples (n = 2) with sample diluent up to 1/10 did not have an impact on the CSF neurogranin TruncP75 concentration when the dilution factor was taken into account (%CV for all concentrations amounted to 3.2 % and 4.3 %).

***Precision****.* Three neat CSF samples (concentrations: 226, 435, 769 pg/mL) were tested in triplicate in 10 different test runs. The % CV, which is a combination of inter-run and intra-run variability, amounted to 7.1, 5.5, and 5.2%, respectively.

**
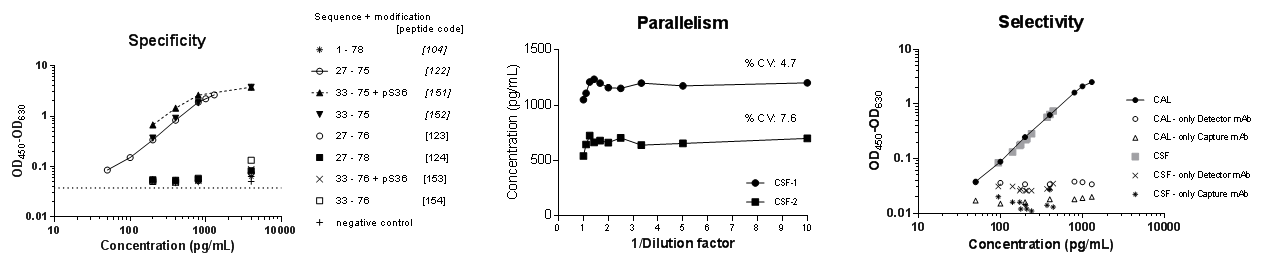
**Figure S3. Analytical performance data for the α-Synuclein ELISA.

***Specificity***. The α-synuclein ELISA is highly specific for the α-isoform of the protein. No cross-reactivity with the β- or γ-synuclein can be detected with the current assay format, not not when using high concentrations. Selected mAbs in the final product are not dependent on phosphorylation of Serine at position 125 or position 129 (unpublished observation).

***Parallelism.*** 4 neat CSF samples were serially diluted (dilution ½ to 1/20) with sample diluent and tested for parallelism. The diluted samples did show a comparable behavior than the calibrators as illustrated by the low variation on recalculated concentration over the dilutions

(between 4.8% and 9.4% CV).


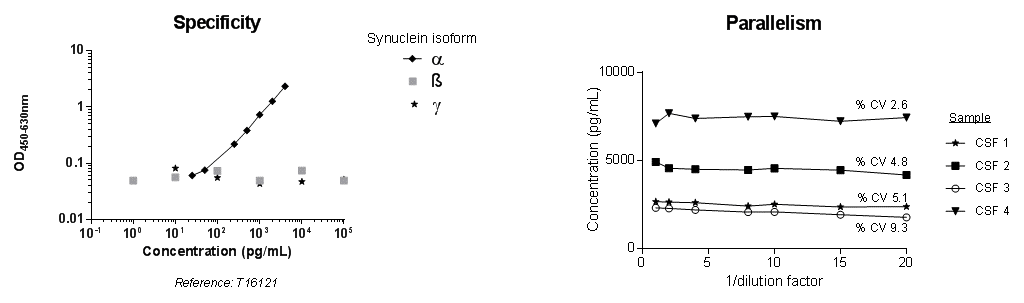
***Precision*.** 5 native pooled CSF samples, with concentrations in the measuring range, were tested in duplicate by two operators in 7 runs in the same laboratory. The between-run (one lab) % CV over the 7 runs ranged between 6.7% and 12.7%.
